# Terminal Ileum Lipoma Causing Ileocolic Intussusception: A Case Report and Literature Review

**DOI:** 10.7759/cureus.49562

**Published:** 2023-11-28

**Authors:** Siddhant Dogra, Jason Wei, Benjamin Wadowski, Virginia Devi-Chou, Leandra Krowsoski, Rajiv R Shah

**Affiliations:** 1 Radiology, NYU Langone Health, New York, USA; 2 Surgery, NYU Langone Health, New York, USA

**Keywords:** intussusception in adults, terminal ileum lipoma, intussusception lead point, ileocecal intussusception, ileocolonic intussusception

## Abstract

Adult intussusception is much rarer than pediatric intussusception and usually occurs secondary to a pathological lead point, most frequently neoplasm. Terminal ileum lipomas are an infrequent cause of adult ileocolic intussusception but can be seen together with the intussusception on initial imaging evaluation, which can guide appropriate diagnosis and management.

We describe a case of a 42-year-old man presenting with 12 hours of severe right lower quadrant pain. CT of the abdomen and pelvis demonstrated an ileocolic intussusception with fat-density lesions within the intussusception as well as in the distal ileum. The patient went to the operating room for laparoscopic ileocolic resection, during which ileo-ileal and ileocolic intussusceptions were identified in the terminal ileum and multiple fatty masses were palpated in the terminal ileum and cecum. Following ileocecectomy, surgical pathology confirmed terminal ileum with intussusception associated with multiple submucosal lipomas.

We also review the literature for cases of ileocolic intussusception caused by terminal ileum lipomas. Patients presented with both acute and chronic symptoms, and while CT was the most common modality used for diagnosis, ultrasound and colonoscopy were also able to identify the intussusception. Although the intussusception was initially reduced in two patients, all patients ultimately underwent surgical resection.

## Introduction

Intussusception, defined as the telescoping of one segment of the gastrointestinal tract into the lumen of an adjacent segment, is much less frequent in adults compared to children [1]. Due to the low incidence and nonspecific symptoms of adult intussusception, clinical diagnosis alone is challenging and imaging is almost always required.

Adult intussusception is often secondary to a pathological lead point, most commonly neoplasm [2]. One potential cause of adult intussusceptions is a terminal ileum lipoma. Although rare, recognizing this entity on imaging is important to ensure proper management.

We present a case of ileocolic intussusception secondary to a terminal ileum lipoma. A review of similar published cases is included to summarize information regarding patient demographics, presenting symptoms, imaging manifestations, and management approaches.

## Case presentation

A 42-year-old man with a history of hepatitis B presented to the emergency department with several hours of severe right lower quadrant pain associated with nausea and vomiting. Physical examination was significant only for tenderness to palpation in the right lower quadrant. No lumps were palpated or visible on examination. Initial laboratory testing was significant only for a leukocytosis of 13.18k/uL.

**Figure 1:**
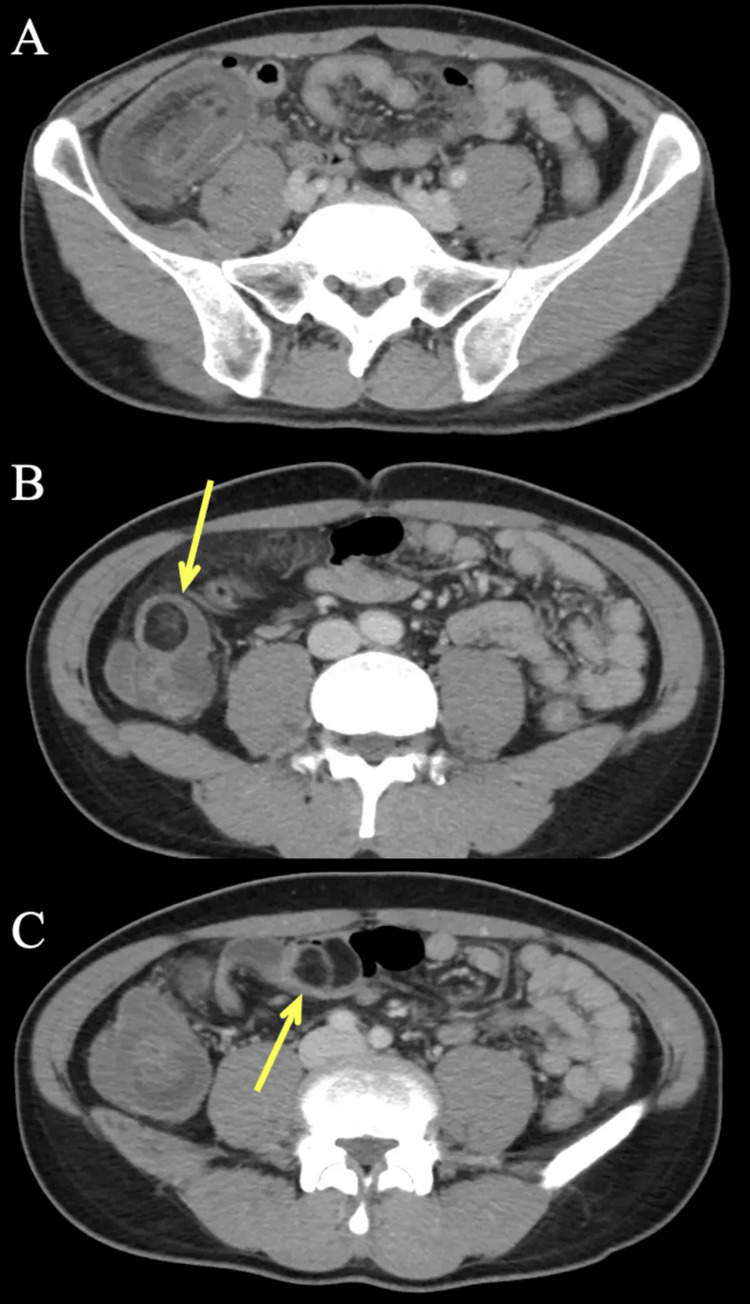
Ileocolic intussusception with terminal ileum lipomas Axial CT abdomen and pelvis slices showing the ileocolic intussusception (A) with terminal ileum lipomas (yellow arrows) both within (B) and outside (C) the intussusception.

CT of the abdomen and pelvis was ordered due to suspected appendicitis (Figure 1).

In the right lower quadrant, the mesenteric fat and vessels were telescoping into the cecum, causing ileocolic intussusception with surrounding inflammatory changes. Multiple fat-density lesions were seen within the intussusception with additional fat-density lesions seen in the terminal ileum. The final impression was ileocolic intussusception likely secondary to terminal ileum lipomas.

General surgery was consulted and the decision was made to proceed with laparoscopic ileocolic resection. In the operating room, a series of ileo-ileal and ileocolic intussusceptions were identified in the terminal ileum and were unable to be laparoscopically reduced. Multiple mobile fatty masses were palpated in the terminal ileum and cecum. As the intussusception was unable to be reduced and there were multiple lesions that could serve as pathologic lead points, a laparoscopic-assisted ileocecectomy with the creation of a side-to-side ileocolic anastomosis was performed. The operative time was approximately five hours, and the estimated blood loss was 25 mL. Surgical pathology confirmed an ileocolic intussusception with early ischemic changes associated with a 4.5 cm submucosal lipoma, with additional submucosal terminal ileum lipomas. The patient had no postoperative complications while in the hospital and tolerated advancement to a low-fiber diet with normal bowel movements. He was discharged on postoperative day four. The patient was progressing well at his one-month post-operative visit.

## Discussion

Lipomas are benign tumors that rarely appear in the intestines, with the reported incidence ranging between 0.15% and 4.4%, more commonly in the large bowel [3]. Most intestinal lipomas are asymptomatic, but those over 2 cm are more likely to cause symptoms or induce an intussusception, as in this case [4].

In order to compare diagnosis, management, and outcomes, we searched PubMed for cases of pathology-confirmed ileocecal intussusception due to a small bowel lipoma serving as a pathologic lead point, published after 2000. We initially found 31 cases; of these, 16 explicitly noted a lipoma in the terminal ileum, defined as within 30 cm proximal to the ileocecal valve [5]. For each, we extracted the age and symptoms at presentation, diagnostic modality, and management (Table 1) [3,4,6-19].

**Table 1:** A literature review of cases of ileocolic intussusception due to terminal ileum lipoma

Authors	Age (years)	Symptoms	Initial Diagnostic Modality	Management
Abbasakoor et al [6]	52	3 months of intermittent abdominal pain and constipation	CT	Laparoscopic resection
Abdulla et al [3]	51	1 day of abdominal pain with vomiting, constipation, and abdominal distension	CT	Laparotomy with resection
Ahmed et al [7]	67	5 days of abdominal pain, vomiting, and obstipation	CT	Laparoscopic resection
Balamoun et al [8]	65	3 days of abdominal pain and bilious vomiting; 10 kg weight loss over the preceding year	CT	Laparotomy with resection
Bilgin et al [4]	39	1-month intermittent abdominal pain, continuous for 24 hours prior to presentation	CT	Laparoscopy converted to open resection
Bosman et al [9]	30	2 days of abdominal pain with nausea and vomiting	Ultrasound	Laparotomy with resection
Krasniqi et al [10]	46	Acute severe abdominal pain with nausea and vomiting	Ultrasound	Laparotomy with resection
Lee et al [11]	29	1 day of epigastric pain, nausea, and fever	CT	Laparoscopic resection
Lee et al [12]	73	2 years of intermittent abdominal pain and weight loss of 15kg	Colonoscopy	Initial reduction with air insufflation, and then colonoscopic polypectomy
Meshikhes et al [13]	55	4 days of epigastric pain	CT	Laparotomy with resection
Nakanishi et al [14]	50	Positive fecal occult blood test	Colonoscopy	Laparoscopy with resection
Namikawa et al [15]	68	Acute severe, colicky lower abdominal pain	Ultrasound	Surgical resection (unclear whether laparoscopic or open)
Shi et al [16]	27	10 days of intermittent abdominal pain	CT	Laparotomy with resection
Shiba et al [17]	33	3 weeks of colicky epigastric pain	CT	Successful barium enema reduction; laparotomy with resection the following day
Tsushimi et al [18]	63	Acute upper abdominal pain and vomiting	Ultrasound	Laparoscopic resection
Wu et al [19]	31	4 years of intermittent abdominal pain	Colonoscopy	Laparoscopic resection

The median age of presentation was 50.5 years (interquartile range 31.5 years-64.5 years), although note that some patients had symptoms for years before presentation. Aside from one patient who was seen for a positive fecal occult blood test, all patients had abdominal pain on presentation. While nine patients presented with acute, severe pain occurring for less than a week, six had been having intermittent pain, sometimes for years, suggesting that these intussusceptions may be intermittent but have a high rate of recurrence if the underlying lipoma is not resected.

Our case shows a classic CT appearance of terminal ileum lipoma causing intussusception, namely, telescoping of segmental bowel contents into a distal segment, displaying a “target sign” and showing distinct, fat-density lesions in the intussusception. Most cases reviewed were also diagnosed on CT, which detects the ileocolic intussusception even if the lipoma is not definitively seen. Many patients initially had abdominal radiographs, which were sometimes suggestive of small bowel obstruction but were non-diagnostic for intussusception. Ultrasound is the diagnostic modality of choice for intussusception in children but was also able to diagnose intussusception in the cases where it was utilized according to our review. Classically, intussusception also shows a "target sign" on ultrasound with alternating hyperechoic and hypoechoic layers. In three cases, the intussusception was directly visualized and diagnosed on colonoscopy, which may be the initial modality utilized for chronic symptoms and could incidentally discover the intussusception and lipomas.

In our case, an ileocecectomy was performed due to the inability to reduce the intussusception. Two of the reviewed cases demonstrated successful reduction, one with air insufflation during colonoscopy and another with barium enema. However, in all cases, including these two, the patient ultimately underwent resection. In one case, the patient initially declined surgery, as her symptoms had improved but was readmitted three months later with recurrent abdominal pain and ultimately underwent resection at that time [15]. Even if symptoms resolve after presentation, the lipomas represent pathological lead points that can cause recurrent intussusception, and resection should be discussed if terminal ileum lipomas are definitively seen on CT.

## Conclusions

Terminal ileum lipomas are rare causes of adult ileocolic intussusception that can present as either chronic intermittent or acute severe abdominal pain. CT and ultrasound can initially identify the underlying intussusception even in adults, though CT more reliably delineates the causative lipomas. The intussusception can be seen on colonoscopy, although it may inadvertently be reduced by the insufflated air, precluding diagnosis. Although reduction is sometimes possible, the high risk of recurrence may warrant resection even if initial symptoms improve.
